# Once-daily S/GSK1349572 combination therapy in antiretroviral-naïve adults: rapid and potent 24-week antiviral responses in SPRING-1 (ING112276)

**DOI:** 10.1186/1758-2652-13-S4-O50

**Published:** 2010-11-08

**Authors:** J Rockstroh, F Felizarta, F Maggiolo, F Pulido, HJ Stellbrink, O Tsybakova, P Yeni, S Almond, C Brothers, I Song, S Min

**Affiliations:** 1 University of Bonn, Bonn-Venusberg, Germany; 2 34th Street Community Health Center, Bakersfield, USA; 3 Ospedali Riuniti de Bergamo, Bergamo, Italy; 4 Hospital 12 de Octubre, Madrid, Spain; 5 ICH Study Center, Hamburg, Germany; 6 AIDS Center, Smolensk, Russian Federation; 7 Hospital Bichat-Claude Bernard, Paris, France; 8 GlaxoSmithKline, Toronto, Canada;; 9 GlaxoSmithKline, Research Triangle Park, USA

## Purpose of study

S/GSK1349572, a next-generation HIV-1 integrase inhibitor, has previously demonstrated potent antiviral activity in Phase 2a with once-daily, unboosted dosing. SPRING-1 is an ongoing dose-ranging study designed to select a dose to for Phase 3 evaluation.

## Methods

SPRING-1 is a Phase 2b, multicentre, partially-blinded study in therapy-naïve adults, randomized 1:1:1:1 to 10mg, 25mg or 50mg of S/GSK1349572 or efavirenz(EFV) 600mg once-daily with either co-formulated TDF/FTC or ABC/3TC.

## Summary of results

205 subjects received study drug: 86% male, 20% non-white, 26%>100,000c/mL HIV-1 RNA, 67% TDF/FTC. Plasma HIV-1 RNA declined rapidly across all S/GSK1349572 doses with no differences in NRTI subgroups. Three protocol-defined virologic failures occurred, 1 on EFV (<1log10 decline by Week 4), and 2 on S/GSK1349572 (Week 4 and 24 rebound >400c/mL with no INI mutation detected). No dose-related clinical or laboratory toxicities were observed. More drug-related AEs of moderate-or-higher intensity were reported on EFV (20%) than S/GSK1349572 (6%) arms; none occurred in more than 1 S/GSK1349572 subject. The most frequent category of such events reported by subjects receiving EFV and S/GSK1349572 were gastrointestinal (4% vs. 2%, respectively); other frequent events on EFV were psychiatric (6%) and rash (4%) disorders. No SAE was considered related to S/GSK1349572. Six subjects (2: S/GSK1349572 and 4: EFV) withdrew due to AEs. Mean change from baseline in LDL cholesterol was +0.023mmol/L among S/GSK1349572 subjects and +0.468mmol/L among EFV subjects. S/GSK1349572 demonstrated low pharmacokinetic variability and drug exposure increased with dose. Table 1

**Table 1:** 

Planned Week 24 Interim Analysis Results	S/GSK1349572 10 mg (n=53)	S/GSK1349572 25mg (n=51)	S/GSK1349572 50mg (n=51)	EFV control (n=50)
Mean baseline HIV-1 RNA (log10 c/mL)	4.42	4.38	4.58	4.46

%<50c/mL at 24 wks (by TLOVR)	96% (51/53)	90% (46/51)	92% (47/51)	78% (39/50)

Median baseline (change from baseline at 24 weeks) CD4+ cells/mm3	289 (+159)	330 (+206)	305 (+167)	308 (+110)†

†p=0.008; Wilcoxon two-sample test vs. S/GSK1349572 arms (median change: +176)

## Conclusions

S/GSK1349572 administered once-daily without a PK booster was well tolerated with potent antiviral activity at all doses explored in SPRING-1. The greater CD4+ cell increases on S/GSK1349572 merit further observation and confirmation.

